# Inhibition of Acid-Sensing Ion Channels by KB-R7943, a Reverse Na^+^/Ca^2+^ Exchanger Inhibitor

**DOI:** 10.3390/biom13030507

**Published:** 2023-03-10

**Authors:** Hua-Wei Sun, Xiang-Ping Chu, Roger P. Simon, Zhi-Gang Xiong, Tian-Dong Leng

**Affiliations:** 1 Neuroscience Institute, Morehouse School of Medicine, Atlanta, GA 30310, USA; 2 Department of Biomedical Sciences, University of Missouri-Kansas City School of Medicine, Kansas City, MO 64108, USA

**Keywords:** ASIC, NCX, KB-R7943, acidosis, stroke, pain

## Abstract

KB-R7943, an isothiourea derivative, is widely used as a pharmacological inhibitor of reverse sodium–calcium exchanger (NCX). It has been shown to have neuroprotective and analgesic effects in animal models; however, the detailed molecular mechanisms remain elusive. In the current study, we investigated whether KB-R7943 modulates acid-sensing ion channels (ASICs), a group of proton-gated cation channels implicated in the pathophysiology of various neurological disorders, using the whole-cell patch clamp techniques. Our data show that KB-R7943 irreversibly inhibits homomeric ASIC1a channels heterologously expressed in Chinese Hamster Ovary (CHO) cells in a use- and concentration-dependent manner. It also reversibly inhibits homomeric ASIC2a and ASIC3 channels in CHO cells. Both the transient and sustained current components of ASIC3 are inhibited. Furthermore, KB-R7943 inhibits ASICs in primary cultured peripheral and central neurons. It inhibits the ASIC-like currents in mouse dorsal root ganglion (DRG) neurons and the ASIC1a-like currents in mouse cortical neurons. The inhibition of the ASIC1a-like current is use-dependent and unrelated to its effect on NCX since neither of the other two well-characterized NCX inhibitors, including SEA0400 and SN-6, shows an effect on ASIC. Our data also suggest that the isothiourea group, which is lacking in other structurally related analogs that do not affect ASIC1a-like current, may serve as a critical functional group. In summary, we characterize KB-R7943 as a new ASIC inhibitor. It provides a novel pharmacological tool for the investigation of the functions of ASICs and could serve as a lead compound for developing small-molecule drugs for treating ASIC-related disorders.

## 1. Introduction

Acid-sensing ion channels (ASICs), members of the degenerin/epithelial sodium channel (Deg/ENaC) superfamily, are expressed throughout the central and peripheral nervous systems [1,2]. At least six ASIC subunits, including ASIC1a, 1b, 2a, 2b, 3, and 4 have been identified [3,4]. Three subunits assemble to form a functional homo- or heterotrimeric channel [5]. ASICs are proton-gated channels sensitive to acidic pH to varying extents depending on the subunit composition of the channels. ASIC1a and 3 are most sensitive to protons with an activation threshold close to pH 7.0. ASIC1a has a pH_0.5_ of ~6.2 and mediates fast decaying, transient currents [6]. ASIC3 can generate biphasic inward currents that contain a transient component with a pH_0.5_ of ~6.2 and a sustained component with a pH_0.5_ of ~4.3 [7,8]. ASIC1b has a similar sensitivity to proton with a pH_0.5_ of ~6.0 [7,9], whereas ASIC2a has a low sensitivity to acidic pH with pH_0.5_ of ~4.4 [10]. ASIC2b and ASIC4 do not form functional homomeric channels [11,12,13]. Activation of ASICs by protons induces sodium influx, resulting in membrane depolarization and neuronal excitation. In addition to sodium, the homomeric ASIC1a and heteromeric ASIC1a + 2b channels are also permeable to calcium [14,15]. Several studies have shown that ASICs play important roles in physiological processes such as synaptic transmission, plasticity, and learning/memory [16,17], and in pathological conditions such as brain ischemia [14,18,19], pain [20,21,22,23], seizure [24], multiple sclerosis [25], Huntington’s and Parkinson’s disease [3], and tumor cell migration [26,27].

A significant tissue pH drop, a condition termed acidosis, is a well-known feature of acute neurological conditions, including brain ischemia, seizure, etc. Acidosis has long been known to aggravate ischemic brain damage; however, the mechanism remained elusive, although a host of possibilities have been suggested. A series of studies have demonstrated a clear link between ASIC1a activation, intracellular calcium accumulation, and acidosis-mediated brain injury [14,15,28]. Pharmacological inhibition of homomeric ASIC1a or heteromeric ASIC1a/2b channels reduces acidosis and ischemia-induced neuronal injury [14,15]. Importantly, blockade of ASIC1a provides a prolonged therapeutic time widow of ~5h for stroke [29], which significantly increases the chances for stroke intervention. In these regards, ASIC1a represents a promising target and the compounds that can inhibit ASIC1a channels may have therapeutic potential for stroke intervention. In addition to brain ischemia, acidosis also occurs in many inflammatory and painful conditions such as skin and muscle incision, arthritis, etc., under which conditions the protons are released by the injured tissues [30,31,32]. It has been demonstrated that the accumulation of protons depolarizes the terminals of nociceptive sensory neurons to cause pain sensation and that the depolarization is caused by direct activation of ASICs [20,21,33]. ASIC1 and ASIC3 have been demonstrated as the leading acid sensors in nociceptors, contributing to acid-induced nociception within a pathophysiologically relevant pH range [20,30,33]. Particularly, the non-desensitizing sustained current component of ASIC3 has been suggested to play a unique role in non-adaptive pain sensation [28,34]. In addition to stroke, ASICs also represent new targets for pain treatment. Compounds that can target ASICs have the potential to be developed as a pain killer.

KB-R7943, an isothiourea derivative, is widely used as a pharmacological inhibitor of reverse sodium–calcium exchanger (NCX) and has been shown to have neuroprotective and analgesic effects [35,36]. However, the underlying mechanism is poorly understood. In the current study, we investigated the effect of KB-R7943 on ASICs to determine whether the inhibition of ASICs might be an underlying mechanism. We first examined the subunit selectivity of KB-R7943 on different homomeric ASICs heterologously expressed in CHO cells. Then, we studied the effect of KB-R7943 on ASIC currents in primary cultured DRG and cortical neurons which express a combination of different homomeric and heteromeric ASICs. In addition, we determined whether the inhibition of ASICs is related to its activity on NCX, and the potential structure–activity relationship by comparing the structurally related NCX inhibitors.

## 2. Materials and Methods

### 2.1. ASICs Transfection in CHO Cells

CHO cells were cultured in F12K medium containing 10% fetal bovine serum (FBS, Invitrogen, Carlsbad, CA, USA), 50 units/mL penicillin, and 50 μg/mL streptomycin. At 50–80% confluence, cells were transfected with cDNA for rat ASIC1a or 2a fused with a green fluorescence protein (GFP) [37], or co-transfected with cDNAs for rat ASIC3 and GFP, as described previously [38]. GFP-positive cells were used for electrophysiological recordings 48–72 h after the transfection.

### 2.2. Primary Culture of DRG Neurons

As described previously [39], DRG neurons were dissected from embryonic Swiss mice at 16 days of gestation (Charles River), enzymatically dissociated with 0.25% trypsin for 10 min, and plated in poly-L-ornithine coated dishes. Cells were initially cultured in DMEM containing 10% FBS and 10% horse serum (HS) at 37 °C in a humidified 5% CO_2_ atmosphere incubator. After 24 h, the culture medium was replaced with Neurobasal medium supplemented with B27 (Invitrogen). The cultures were fed twice a week and used for electrophysiological recordings 6~8 days after plating.

### 2.3. Primary Culture of Mouse Cortical Neurons

Mouse cortical neurons were isolated and cultured as described in our previous study with modification [40]. The use of mice for neuronal cultures was approved by the Institutional Animal Care and Use Committee of Morehouse School of Medicine. Briefly, the brains of fetuses (embryonic day 16) were removed quickly from pregnant mice following anesthesia and cervical dislocation, and placed in cold Ca^2+^/Mg^2+^-free PBS. Cerebral cortices were dissected and incubated with 0.05% trypsin-EDTA for 10 min at 37 °C, followed by trituration. Cells were plated in poly-L-ornithine-coated culture dishes. Cells were initially cultured in minimal essential medium (MEM) with 10% FBS, 10% HS, and 25 mM glucose at 37 °C in a humidified 5% CO_2_ atmosphere incubator for 24 h. Then, the culture medium was completely replaced by a Neurobasal medium supplemented with B-27 and changed twice a week. Neurons were used for the experiments between days 10 and 14.

### 2.4. Electrophysiology

Whole-cell ASIC currents were recorded using a combination of patch-clamp and fast perfusion techniques, as described previously [39]. GFP-positive CHO cells were selected for the recordings of ASIC currents. For fast perfusion, a multibarrel perfusion system (SF-77B, Warner Instruments, Hamden, CT) was used. Patch pipettes were pulled from borosilicate glass. Pipettes had a resistance of 2–4 MΩ when filled with the intracellular solution. Currents were recorded using Axopatch 200B amplifiers (Axon Instruments, Foster City, CA, USA). All data were filtered at 2 kHz and digitized at 5 Hz using Digidata 1320 DAC units (Axon Instruments). Only recordings with an access resistance of less than 10 MΩ and a leak current of less than 100 pA at −60 mV were included for data analysis [40]. The maximal inward current value was measured as the peak current. The sustained current component of ASIC3 was measured at the end of the 4 sec perfusion of acidic solutions. Since ASIC1a currents show significant run-down in the first ~15 min of whole-cell recording, in general, the effect of KB-R7943 (Sigma-Aldrich, Inc, St. Louis, MO, USA) was tested ~20 min after the initiation of whole-cell configuration and following the recording of at least three stable ASIC currents.

### 2.5. Solutions and Chemicals

The extracellular solution contained (mM): 140 NaCl, 5.4 KCl, 20 HEPES, 10 Glucose, 2 CaCl_2_, and 1 MgCl_2_; the pH was adjusted to selected levels with NaOH and HCl, and 320–330 mOsm. The intracellular solution contained (mM): 140 CsF, 1 CaCl_2_, 10 HEPES, 11 EGTA, 2 TEA, 4 MgCl_2_, pH 7.3, adjusted with CsOH, and 290–300 mOsm [40]. KB-R7943 (CAS No.: 182004-64-4) was purchased from Sigma-Aldrich (St. Louis, MO) and has a purity of ≥98% (HPLC). DMSO was used to dissolve the compound to make a 100 mM stock solution, which was kept at −20 °C until use.

### 2.6. Statistical Analysis

All data are expressed as mean ± SEM. Statistical analyses were performed by ANOVA using GraphPad Prism 9. *p* < 0.05 was considered as statistically significant.

## 3. Results

### 3.1. KB-R7943 Use-Dependently Inhibits ASIC1a Currents in CHO Cells

**Figure 1:**
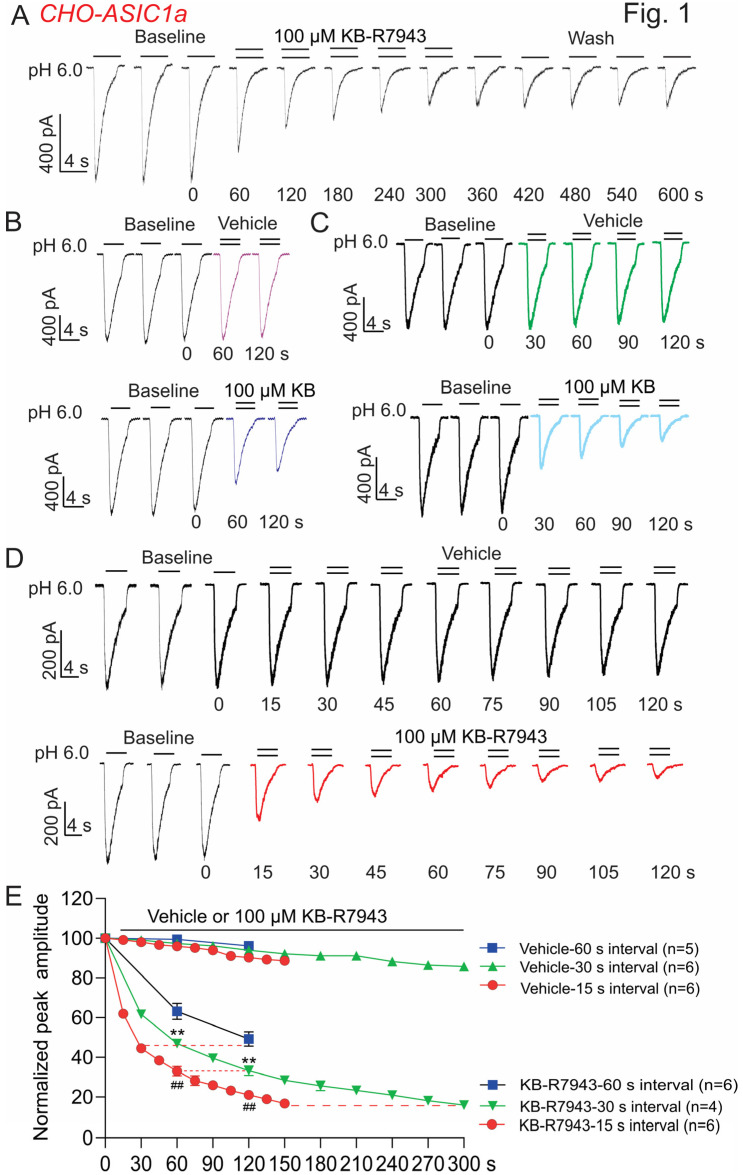
KB-R7943 inhibits homomeric ASIC1a currents in CHO cells (CHO-ASIC1a). ASIC1a was transiently expressed in CHO cells and the currents were recorded at 48–72 h after transfection. (**A**) Representative current traces show a time-dependent and irreversible inhibition of the ASIC1a currents by KB-R7943. KB-R7943 (100 μM) was added in the pH 6.0 solution. ASIC1a currents were induced by a pH drop from 7.4 to 6.0 and the effect of KB-R7943 was not tested until at least 3 consecutive stable currents were obtained. (**B**–**D**) Representative current traces show that KB-R7943 inhibits ASIC1a currents at 3 different stimulating frequencies with intervals of 60, 30, and 15 s between activations of ASIC1a. (**E**) Summary data show that KB-R7943 inhibits more ASIC1a currents at higher-frequency stimulation (*n* = 4–6, ** *p* < 0.01 compared with 60 s interval, ^##^ *p* < 0.01 compared with 30 s interval, ANOVA followed by Turkey’s multiple comparisons test). Data were expressed as mean ± SEM.

We first examined whether KB-R7943 affects ASIC1a currents. ASIC1a is transiently expressed in CHO cells (CHO-ASIC1a) as described previously [39]. At 48–72 h after transfection, ASIC1a currents were induced by a pH drop from 7.4 to 6.0. Considering the run-down of ASIC1a currents [41], the effect of KB-R7943 was not tested until at least three consecutive stable currents were obtained. KB-R7943 (100 μM) was added in pH 6.0 solution. It inhibits ASIC1a currents in a time-dependent manner, and the inhibition is irreversible after washout for 5 min (Figure 1A). The time-dependent reduction of ASIC1a currents suggests a potential mechanism of use-dependent inhibition. Use-dependent inhibition often refers to frequency-dependent inhibition [42], i.e., within the same period of time; the higher the frequency of channel activation in the presence of an inhibitor, the more inhibition takes place. To test this possibility, three stimulating frequencies with an interval of 60, 30, and 15 s between activations of ASIC1a were used (Figure 1B–D). As expected, we found that, within the same period of 120 s, the higher frequency of stimulation causes more ASIC1a currents inhibited by KB-R7943 (Figure 1E). KB-R7943 inhibits 78%, 67%, and 51% of the ASIC1a currents under high-frequency stimulation (15 s interval), medium-frequency stimulation (30 s interval), and lower-frequency stimulation (60 s interval), respectively (Figure 1E, *n* = 4–6, ANOVA, * *p* < 0.05, ** *p* < 0.01 compared with 60 s interval, ^##^ *p* < 0.01 compared with 30 s interval).

**Figure 2:**
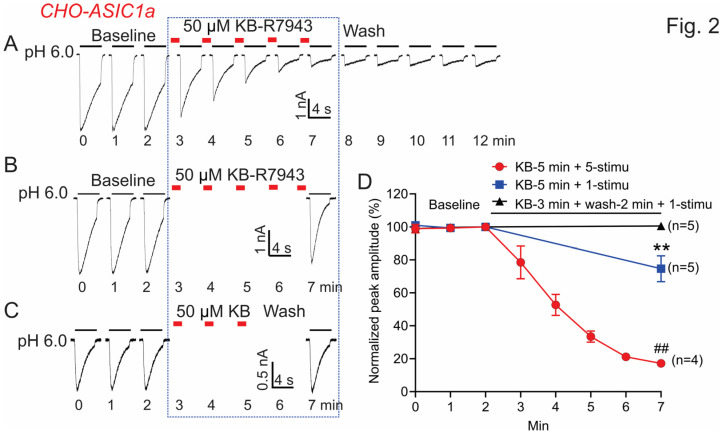
Application of KB-R7943 in pH 7.4 solution inhibits homomeric ASIC1a currents in CHO cells (CHO-ASIC1a). ASIC1a was transiently expressed in CHO cells and the currents were recorded at 48–72 h after transfection. (**A**) Representative current traces show a time-dependent and irreversible inhibition of the ASIC1a currents by KB-R7943. KB-R7943 (50 μM) was added in the pH 7.4 solution. ASIC1a currents were induced by a pH drop from 7.4 to 6.0 and the effect of KB-R7943 was not tested until at least 3 consecutive stable currents were obtained. (**B**) Representative current traces show the ASIC1a currents after 5 min perfusion of KB-R7943 with one stimulus of the ASIC1a channels. KB-R7943 was not administered during the 4S of switching the barrel without acidic solution perfusion. (**C**) Representative current traces show the ASIC1a currents after 3 min perfusion of KB-R7943 followed by a 2 min wash. KB-R7943 was not administered during the 4S of switching the barrel without acidic solution perfusion. (**D**) Summary data show that KB-R7943 use-dependently inhibits ASIC1a currents (*n* = 4–5, ** *p* < 0.01 compared with 3 min perfusion of KB-R7943 followed by a 2 min wash; ^##^ *p* < 0.01 compared with 5 min perfusion of KB-R7943 plus one stimulus of the ASIC1a channels. ANOVA followed by Turkey’s multiple comparisons test). Data were expressed as mean ± SEM.

In addition, we also examined whether KB-R7943 inhibits ASIC1a currents when added to the pH 7.4 solution. We first tried 100 μM and found that this concentration causes an unstable recording after 2 to 3 min perfusion (data not shown), which makes the analysis unreliable. We then changed to a lower concentration of 50 μM and observed a significant “time-dependent” inhibition, which is irreversible. Around 80% of the ASIC1a currents are inhibited by 50 μM KB-R7943 after 5 min perfusion with 5 stimuli of the channel (Figure 2A,D). To determine whether this inhibition is use-dependent, we compared the extent of inhibition between 1 and 5 stimuli in the presence of 50 μM KB-R7943, within the same period of 5 min. Our data show that only ~20% of the currents are inhibited by 5 min perfusion of KB-R7943 with 1 stimulus, but ~80% of the currents are inhibited by 5 min perfusion of KB-R7943 with 5 stimuli (Figure 2A,B,D). These data together with the data in Figure 1 strongly suggest that KB-R7943 inhibits ASIC1a channel in a use-dependent manner. Considering the fact that KB-R7943 use-dependently and irreversibly inhibits ASIC1a currents when it is only present in the pH 7.4 solution, we speculate that KB-R7943 could also bind with the inactivated channels near the channel pore region and when the channels are opened it gets trapped in the channel pore, which makes it difficult to be washed out. The binding on the inactivated channel might be loose and easy to be washed away since it is not trapped in the channel pore yet. To test this hypothesis, we examined the ASIC1a currents after perfusion of KB-R7943 in the pH 7.4 solution for 3 min followed by a 2 min wash out. We found that there is no significant change in the amplitude of the ASIC1a currents (Figure 2C–D), suggesting that KB-R7943 can be washed away easily from the inactivated ASIC1a.

### 3.2. KB-R7943 Inhibits ASIC1a Currents in a Concentration-Dependent and pH-Independent Manner in CHO Cells

**Figure 3:**
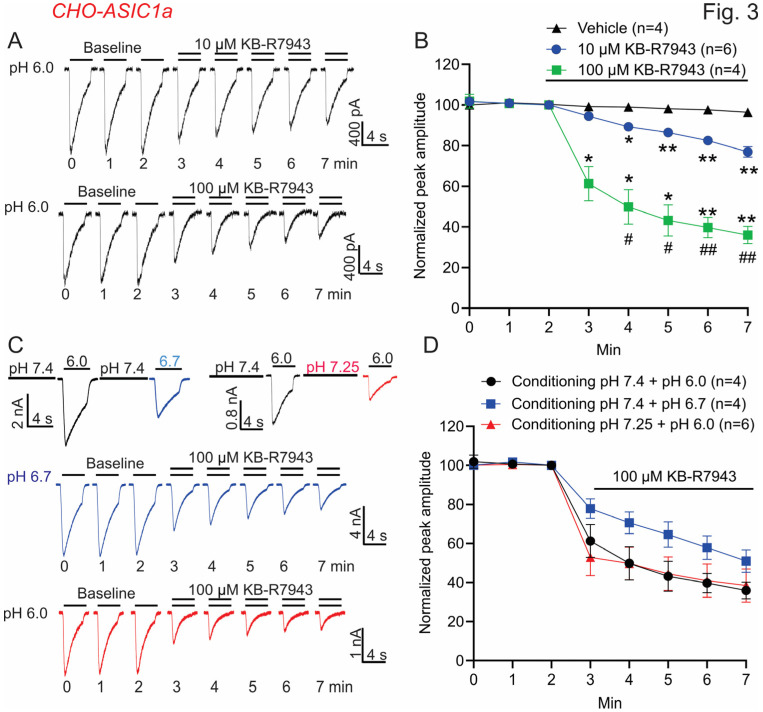
Concentration-dependent and pH-independent inhibition of homomeric ASIC1a currents by KB-R7943 in CHO cells (CHO-ASIC1a). (**A**,**B**). Representative current traces and summary data show the inhibition of the ASIC1a currents by 10 μM and 100 μM KB-R7943. KB-R7943 was only added in pH 6.0 solution. ASIC1a currents were induced by a pH drop from 7.4 to 6.0 and the effect of KB-R7943 was not tested until at least 3 consecutive stable currents were obtained, *n* = 4–6, ANOVA, * *p* < 0.05 and ** *p* < 0.01 compared with the vehicle; ^#^ *p* < 0.05 and ^##^ *p* < 0.01 compared with 10 μM KB-R7943. (**C**) The upper left panel shows the ASIC1a currents activated at different pHs of 6.0 and 6.7 with the same conditioning pH of 7.4. The upper right panel shows the ASIC1a currents activated at pH 6.0 with different conditioning pHs of 7.4 and 7.25. The middle panel shows that KB-R7943 inhibits the ASIC1a currents activated at pH 6.7 with the conditioning pH of 7.4. The lower panel shows that KB-R7943 inhibits the ASIC1a currents activated at pH 6.0 with the conditioning pH of 7.25. KB-R7943 was only added in pH 6.7 and 6.0 solutions. (**D**) Summary data show that KB-R7943 inhibits ASIC1a currents at different activation and conditioning pHs (*n* = 4–6). Data were expressed as mean ± SEM.

We further determined the concentration and pH dependence of KB-R7943 on ASIC1a currents. First, we compared the inhibitory effect of 10 μM and 100 μM KB-R7943 on ASIC1a currents with 5 times of stimulation of the channels. As shown in Figure 3A,B, KB-R7943 inhibits ASIC1a currents in a concentration-dependent manner: at the end of 5 times of ASIC1a activation, around 23% and 64% of the currents are inhibited by 10 μM and 100 μM KB-R7943, respectively (Figure 3A,B, *n* = 4–6, ANOVA, * *p* < 0.05 and ** *p* < 0.01 compared with the vehicle; ^#^ *p* < 0.05 and ^##^ *p* < 0.01 compared with 10 μM KB-R7943). Then, we determined whether KB-R7943 exerts its inhibition in a pH-dependent manner. We first compared the inhibitory effect of 100 μM KB-R7943 on ASIC1a currents activated at different pH levels of 6.0 and 6.7. We found that KB-R7943 exerts a similar inhibition of the ASIC1a currents activated at pH 6.7 (Figure 3C, middle panel) to that activated at pH 6.0 (Figure 3A, lower panel), without statistical significance (Figure 3D). We then compared the inhibitory effect of 100 μM KB-R7943 on ASIC1a currents activated at pH 6.0 with different conditioning pH levels of 7.4 and 7.25. KB-R7943 exerts a similar inhibition of the ASIC1a currents at both conditioning pH levels of 7.25 (Figure 3C, lower panel) and 7.4 (Figure 3A, lower panel), without statistical significance (Figure 3D). These data suggest that KB-R-7943 might inhibit ASIC1a in a pH-independent manner.

### 3.3. Effect of KB-R7943 on Homomeric ASIC2a and ASIC3 in CHO Cells

**Figure 4:**
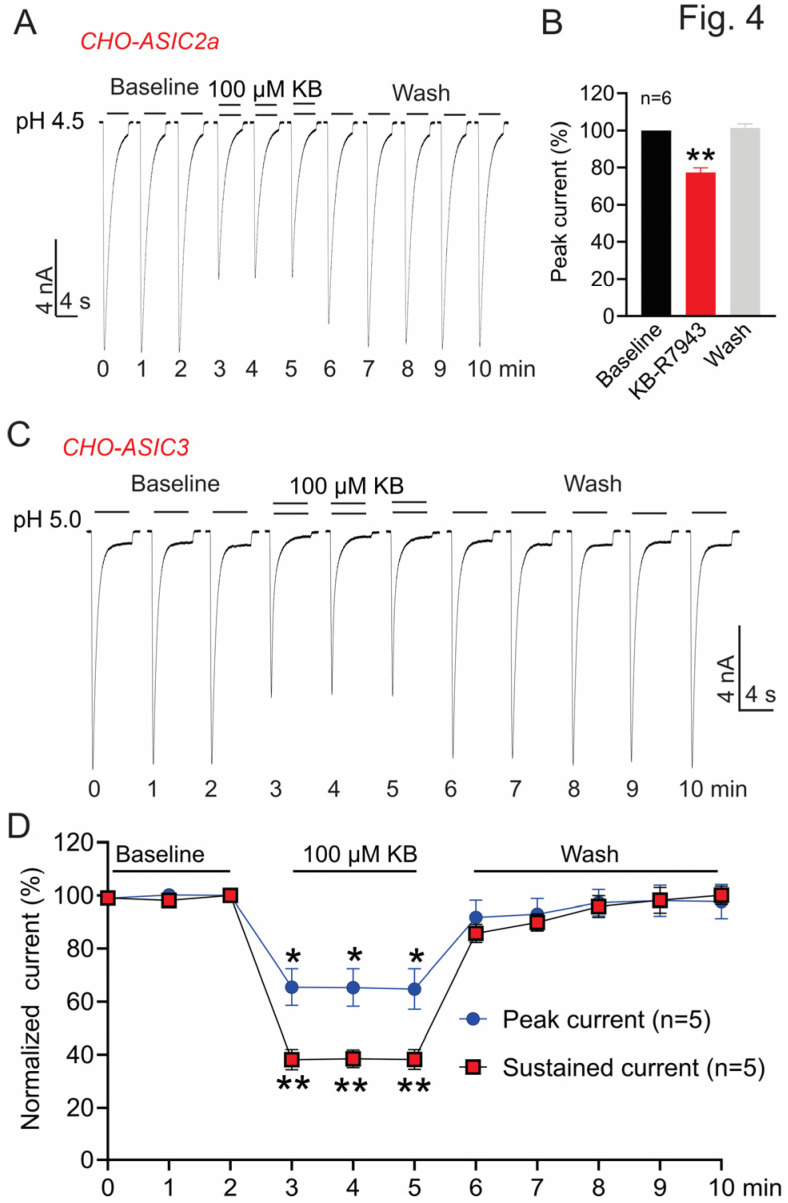
KB-R7943 Inhibits homomeric ASIC2a and ASIC3 currents in CHO cells (CHO-ASIC2a and CHO-ASIC3). ASIC2a and ASIC3 were transiently expressed in CHO cells and the currents were recorded at 48–72 h after transfection. (**A**) Representative current traces show that KB-R7943 (100 μM) rapidly and reversibly inhibits ASIC2a currents. ASIC2a currents were induced by pH 4.5 acidic solution. (**B**) Summary data show the normalized peak current amplitudes immediately before, at the end of KB-R7943 application and at the end of washout (*n* = 6, ** *p* < 0.01 compared with the baseline values immediately before KB-R7943 application, ANOVA). (**C**) Representative current traces show that KB-R7943 rapidly and reversibly inhibits ASIC3 currents. The biphasic ASIC3 currents were induced by pH 5.0 acidic solution. (**D**) Summary data show that KB-R7943 inhibits the transient (blue) and sustained (red) ASIC3 currents (*n* = 5, * *p* < 0.05 and ** *p* < 0.01 compared with the baseline values immediately before KB-R7943 application, ANOVA, followed by Dunnett’s multiple comparisons test).

To determine whether the inhibition of KB-R7943 on ASICs is subunit-dependent, we also examined its effect on homomeric ASIC2a and ASIC3 channels. ASIC2a is present in both central and peripheral nervous systems, which can form homomeric ASIC2a and heteromeric channels with other ASIC subunits. Unlike homomeric ASIC1a which has a high sensitivity to proton (pH_0.5 act_ ~6.0), homomeric ASIC2a has a relatively low sensitivity to acidic pH (pH_0.5 act_ ~4.5) [9,43]. To activate ASIC2a, we used a perfusion solution with pH 4.5. The effect of KB-R7943 was tested after three stable current traces were obtained. Our data show that, unlike its use-dependent and irreversible inhibition on ASIC1a, KB-R7943 (100 μM) exhibits a fast and reversible inhibition on ASIC2a. The inhibition reaches a steady state rapidly and is washed back immediately (Figure 4A). At the end of the KB-R7943 application, ~23% of the peak ASIC2a currents are inhibited (Figure 4B, *n* = 6, ** *p* < 0.01 compared with the baseline value immediately before KB-R7943 application). Next, we examined whether KB-R7943 affects ASIC3, which is abundantly and restrictedly expressed in the peripheral nervous system [8,9]. ASIC3 can generate two current components including the transient and sustained currents. The sustained current component has a relatively lower sensitivity to protons [7,8], which can be induced when the pH drops below 5 (Figure 4C). Similar to its effect on ASIC2a currents, KB-R7943 (100 μM) rapidly and reversibly inhibits ASIC3 currents (Figure 4C). It inhibits ~35% of the transient current and ~62% of the sustained current (Figure 4D, *n* = 5, * p < 0.05 and ** p < 0.01 compared with the baseline value immediately before KB-R7943 application).

### 3.4. KB-R7943 Inhibits ASICs in Primary Cultured DRG Neurons

**Figure 5:**
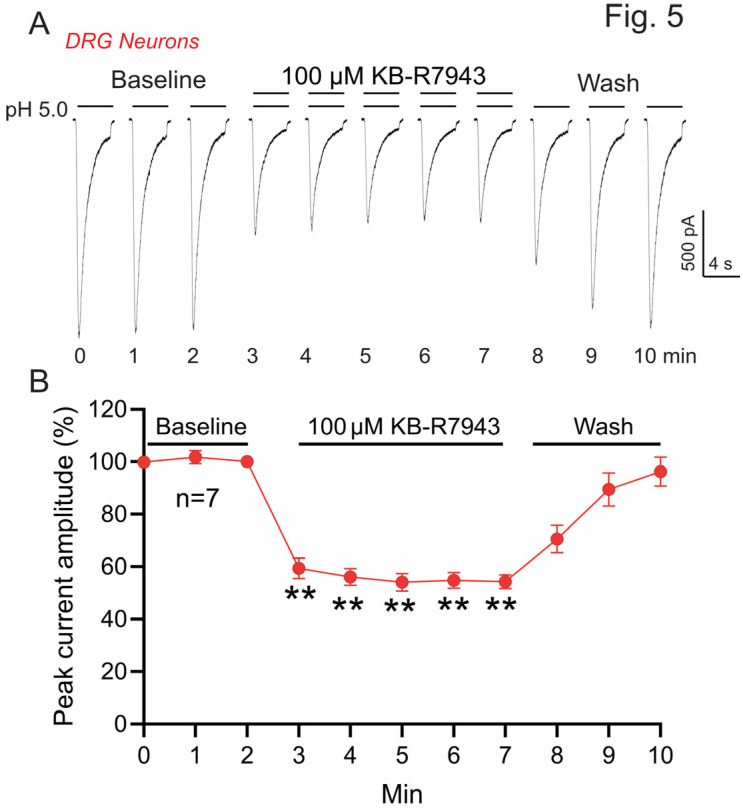
KB-R7943 inhibits ASIC-like currents in mouse dorsal root ganglion (DRG) neurons. (**A**) Representative current traces show that KB-R7943 (100 μM) reversibly inhibits the ASIC-like currents in DRG neurons. ASIC-like currents were induced by a pH 5.0 acidic solution. (**B**) Summary data show that KB-R7943 inhibits the peak ASIC-like currents (*n* = 7, ** *p* < 0.01 compared with the baseline values immediately before KB-R7943 application, ANOVA, followed by Dunnett’s multiple comparisons test).

Heterologous expression has proven to be a powerful tool to elucidate the functional properties of ion channels. However, it has limitations, for example, it cannot fully mimic the ratio of different subunits and complicated subtypes of ion channels in the native cells. In this regard, we further examined the effect of KB-R7943 on ASIC currents in primary cultured DRG and cortical neurons, which express a mixture of homomeric and heteromeric ASICs. The effect of KB-R7934 on ASIC currents mediated by different ASIC subunits in CHO cells suggests that it might affect the ASICs in the central and peripheral neurons. To test this hypothesis, we first examined the effect of KB-R7934 on ASICs in primary cultured mouse DRG neurons, which express ASIC1a, 1b, 2a, 2b, and 3. ASIC3 is the major ASIC subunit expressed in DRG neurons [44], which can form homomeric and heteromeric ASIC3 channels. ASIC-like current was induced by perfusing acidic solution at pH 5.0. The currents are significantly and reversibly inhibited by KB-R7943 (100 μM) (Figure 5A). The peak current is inhibited by ~51% (Figure 5B, *n* = 7, ** *p* < 0.01 compared with the baseline value).

### 3.5. KB-R7943 Inhibits ASICs in Primary Cultured Mouse Cortical Neurons

**Figure 6:**
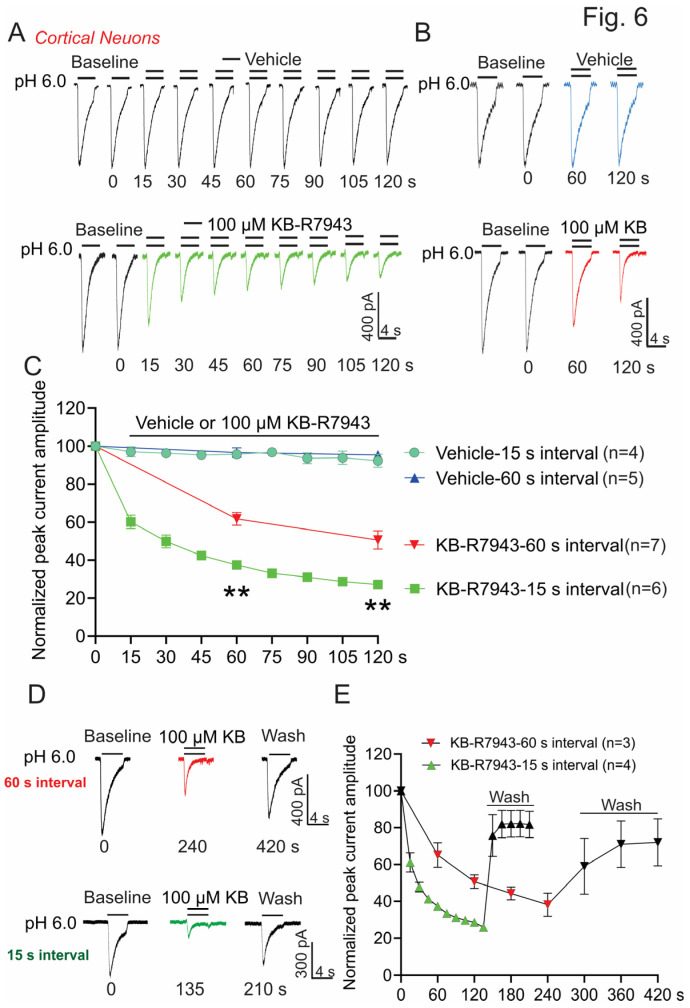
KB-R7943 inhibits ASIC1a-like currents in mouse cortical neurons. (**A**,**B**) Representative current traces show that KB-R7943 (100 μM) inhibits the ASIC1a currents at different stimulating frequencies, including 15 s and 60 s intervals, respectively. (**C**) Summary data show that KB-R7943 inhibits more ASIC1a currents at higher-frequency stimulation (15 s interval) (*n* = 4–7, ** *p* < 0.01 compared with 60 s interval, ANOVA, followed by Turkey’s multiple comparisons test). (**D**,**E**) Representative current traces show that KB-R7943 (100 μM) reversibly inhibits the ASIC1a currents at 60 s and 15 s intervals, respectively.

Next, we determined the effect of KB-R7943 on ASICs in central neurons. The primary cultured mouse cortical neurons were used. ASIC1a is the major ASIC subunit expressed in the brain neurons, which can form homomeric ASIC1a or heteromeric channels with ASIC2a or 2b [15]. These heteromeric channels generate similar ASIC currents as homomeric ASIC1a that are difficult to be distinguished from each other. KB-R7943 (100 μM) inhibits the ASIC1a-like currents in cortical neurons in a time-dependent manner (Figure 6A). Again, the time-dependent decrease in ASIC1a currents by KB-R7943 suggests a use-dependent inhibition. To test this possibility, we examined the inhibitory effect of KB-R7943 on ASIC1a-like currents under two different stimulating frequencies with 15 s and 60 s intervals (Figure 6A,B). Our data show that, within the period of 2 min, KB-R7943 inhibits ~73% of the ASIC1a-like currents at the high-frequency stimulation (15s interval), whereas only ~49% of the currents are inhibited at the low-frequency stimulation (60 s interval), suggesting a use-dependent inhibition (Figure 6C, *n* = 4–7, ANOVA, ** *p* < 0.01 compared with 60 s interval). We performed washout in some neurons after KB-R7943 application and found that the currents are largely reversible, which recover to ~70 to 80% of the original values (Figure 6D,E, *n* = 3–4).

### 3.6. The Effect of KB-R7943 on ASICs Is Independent of NCX Inhibition

**Figure 7:**
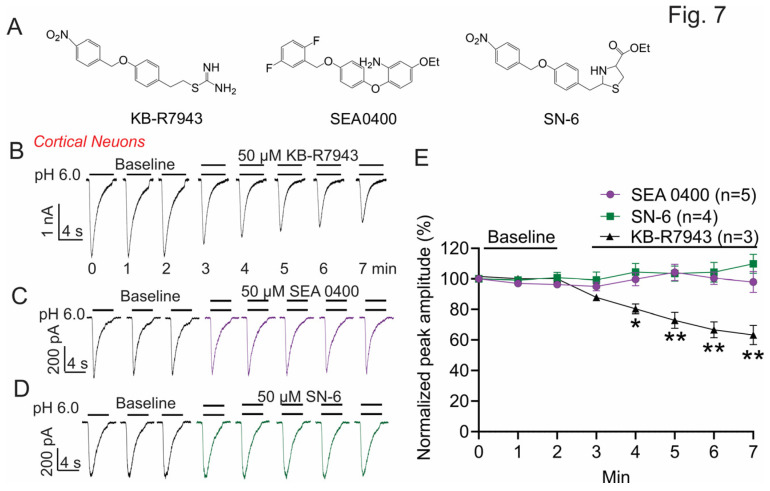
Effect of KB-R7943 and analogs on ASIC1a-like currents in mouse cortical neurons. (**A**) The structures of KB-R7943 and its analogs, including SEA-0400 and SN-6. (**B**–**D**) The effect of KB-R7943 (50 μM), SEA0400 (50 μM), or SN-6 (50 μM) on ASIC1a-like currents. (**E**) Summary data show the effect of KB-R7943, SEA0400, and SN-6 on ASIC1a-like currents (*n* = 3–5, * *p* < 0.05 and ** *p* < 0.01 compared with the baseline value immediately before KB-R7943 application, ANOVA, followed by Dunnett’s multiple comparisons test).

KB-R7943 is widely used as a reverse NCX inhibitor. To determine whether the effect of KB-R7943 on ASICs is coupled with its effect on NCX, we examined the potential effect of other NCX inhibitors, including SEA0400 and SN-6, on ASIC1a-like currents in cortical neurons (Figure 7A). The IC_50_ on NCX is 5.7 μM for KB-R7943 [45], 2.9–16 μM for SN-6 [46], and 33 nM for SEA0400 [47]. We tested these compounds at a high concentration of 50 μM which should maximally inhibit the NCX activity. We found that only KB-R7943 has a significant inhibitory effect on ASIC currents (Figure 7B,E). Neither SEA0400 nor SN-6 show any effect (Figure 7C–E). These data suggest that the inhibitory effect of KB-R7943 on ASICs is independent of its activity on NCX. In addition, these findings also suggest that the isothiourea group, which is only present in KB-R7943, may play an important role.

## 4. Discussion

In the present study, we demonstrated that KB-R7943 negatively modulates ASICs. It inhibits the homomeric ASIC1a in a use- or frequency-dependent manner; the higher the frequency of channel activation within a fixed period of time, the greater the inhibition occurs. The inhibition is pH independent. Some ASICs inhibitors exert their inhibition by shifting either the pH dependence of activation to a more acidic pH or that of steady-state desensitization (inactivation) to more alkaline pH. For example, PcTx-1 inhibits ASIC1a by shifting the steady-state desensitization curve of ASIC1a to an alkaline pH [48]. In contrast, the change of the conditioning or activation pH dose not significantly affect the inhibitory effect of KB-R7943. In addition, the inhibition is irreversible, which might be caused by a tight binding that makes KB-R7943 difficult to wash away, or an altered conformation of the ASIC1a upon KB-R7943 binding traps it deeply in certain locations, e.g., the channel pore, resulting in difficulty for washout. In contrast to ASIC1a, the homomeric ASIC2a and ASIC3 channels are reversibly inhibited by KB-R7943. Both the transient and sustained current components of ASIC3 are inhibited. Furthermore, KB-R7943 inhibits the ASIC-like currents in DRG neurons and the ASIC1a-like currents in the cortical neurons. The inhibition of ASIC1a-like currents is also use-dependent and is unrelated to its effect on NCX since neither of the other two well-characterized NCX inhibitors shows an effect on ASIC. The inhibition might be dependent on the isothiourea group, which is present in KB-R7943 but not in other structurally related analogs including SEA0400 and SN-6. Interestingly, even though amiloride and other analogs have a similar functional group of guanidine, none of these compounds can inhibit ASIC like KB-R7943 [49]. Their effect on ASIC1a is fast and reversible [49]. These differences indicate that the isothiourea group determines the unique pharmacological properties of KB-R7943 on ASICs.

ASICs are predominantly expressed in neurons. The different ASIC subunits have distinct expression profiles. ASIC1a, 2a, and 2b are expressed in both the central and peripheral nervous system, while ASIC1b and 3 are restricted to the peripheral nervous system [8,9,50]. ASICs are trimeric channels, which can be either homomeric that are composed of the same subunits, or heteromeric channels that are composed of at least two different types of subunits. For example, the peripheral neurons may also express homomeric ASIC1a, 1b, and 3 channels, or heteromeric ASICs containing different combinations of these subunits [37,51]. Similarly, the central neurons can express homomeric ASIC1a and 2a channels, as well as heteromeric ASICs such as ASIC1a/2a, ASIC1a/2b, etc. [15]. Some homomeric and heteromeric channels share similar electrophysiological properties that cannot be distinguished from each other due to the lack of pharmacological tools. In this regard, the inhibited ASIC-like currents in DRG neurons and ASIC1a-like currents in cortical neurons by KB-R7943 could be mediated by either homomeric or heteromeric ASICs.

The inhibition of the ASIC-like currents in DRG neurons is completely reversible, suggesting an extremely low level of homomeric ASIC1a channels in these neurons because the inhibition of the homomeric ASIC1a by KB-R7943 is irreversible. This is consistent with the previous studies which suggest that the heterotrimeric ASIC3 is the leading form of ASICs in the peripheral neurons [22,52]. ASIC3 is largely colocalized with ASIC2b in sensory neurons to form a heteromeric channel in DRG neurons, which can also produce a biphasic current similar to that of the homomeric ASIC3 [44,53]. Based on these facts, we speculate that the inhibited ASIC-like currents by KB-R7943 are likely mediated by a combination of homomeric ASIC3 and heteromeric ASIC3, e.g., ASIC3/2b. ASICs, particularly ASIC3, have been implicated in a variety of pain sensations [21,54,55]. The sustained non-desensitizing current component can produce a long-lasting depolarization of the neuronal membrane of the nociceptors, contributing to persistent pain [8,54,56]. Intriguingly, the widely used small molecule ASIC inhibitor amiloride potentiates this sustained ASIC-like current component in sensory neurons [34]. This may explain why amiloride was less effective in reducing acid-evoked pain under more severe acidification conditions when the sustained currents are evoked compared with that under moderate acidification conditions (pH ≥ 6.0) when only the transient currents are evoked [23,39]. In contrast, KB-R7943 inhibits both current components, suggesting that it may have therapeutic potential for relieving pain under a broader pH range of acidic conditions. A recent study has reported that KB-R7943 exerts antinociceptive activity in rodent neuropathic pain model [35], and inhibition of ASICs might be an important mechanism.

KB-R7943 inhibits ASIC1a-like currents in primary cultured mouse cortical neurons in a use-dependent manner. The recorded ASIC1a-like currents are generated by the activation of a mixture of homomeric and heteromeric ASIC1a channels, which are difficult to be distinguished from each other. The inhibition is largely reversible, suggesting that most of the inhibited currents might be mediated by the heteromeric ASIC1a channels, since the inhibition of homomeric ASIC1a by KB-R7943 is irreversible. A previous report suggests that, in primary cultured brain neurons, ~20% ASICs are homomeric ASIC1a, ~30% are ASIC1a/2a, and the majority of ASIC channels (~50%) are ASIC1a/2b [15]. KB-R7943 inhibits >70% of the ASICs in primary cultured cortical neurons, suggesting that it may inhibit the heteromeric ASIC1a channels, e.g., ASIC1a/2a or ASIC1a/2b. Regarding the fact that activation of homomeric ASIC1a and/or heteromeric ASIC1a/2b contributes to ischemia and acidosis-induced neuronal damage [14,15], we speculate that the inhibition of ASIC1a or possible ASIC1a/2b by KB-R7943 might be an important component of the mechanism underlying its neuroprotective activity [36]. In the future, the combination of specific ASIC1a inhibitor PcTx1 and ASIC1a knockout mice could yield data that provide further confirmation of this mechanism.

In summary, we have revealed that KB-R7943 is a novel ASIC inhibitor. This provides a new pharmacological tool for investigating ASICs functions. In addition, our results provide an important lead compound for developing ASIC inhibitors for treating ASIC-related neurological disorders. Future structural modification may help identify a new compound with a specific inhibitory effect on ASIC without affecting NCX, which could be a drug candidate for therapeutic applications.

## Data Availability

The datasets used and/or analyzed during the current study are available from the corresponding author upon reasonable request.
